# TLRs, Alcohol, HCV, and Tumorigenesis

**DOI:** 10.1155/2010/518674

**Published:** 2011-02-09

**Authors:** Keigo Machida

**Affiliations:** Department of Molecular Microbiology and Immunology, 503B-HMR, University of Southern California Keck School of Medicine, Los Angeles, CA 90033, USA

## Abstract

Chronic liver damage caused by viral infection, alcohol, or obesity can result in increased risk for hepatocellular carcinoma (HCC). Ample epidemiological evidence suggests that there is a strong synergism between hepatitis C virus (HCV) and alcoholic liver diseases (ALD). The Toll-like receptor (TLR) signaling pathway is upregulated in chronic liver diseases. Alcoholism is associated with endotoxemia that stimulates expression of proinflammatory cytokine expression and inflammation in the liver and fat tissues. Recent studies of HCC have centered on cancer-initiating stem cell (CSC), including detection of CSC in cancer, identification of CSC markers, and isolation of CSC from human HCC cell lines. Synergism between alcohol and HCV may lead to liver tumorigenesis through TLR signaling.

## 1. HCV, Alcohol, and HCC

Chronic liver damage caused by viral infection, alcohol, or metabolic syndrome can result in increased risk for HCC which is the third most common cancer in the world [1]. This virus is a major cause of HCC, which is the fifth most common cancer in the world. HCC has a low five-year survival rate due to the lack of therapeutic options and is highly prevalent in the world, especially in Africa and Asia [1]. Clearly, understanding the molecular mechanisms of HCV-induced hepatocarcinogenesis is required for the eventual development of improved therapeutic modalities for this disease [2]. In particular, chronic infection with HBV or HCV represents a major risk factor for HCC [1]. HCV affects more than 170 million people worldwide [1, 3, 4]. 

Ample epidemiological evidence suggests that there is a strong connection between hepatitis C virus (HCV) and ALD. First, the prevalence of HCV is significantly higher among alcoholics than in the general population; for example, while the HCV positive rate in the general population of the U.S. is roughly 1%, it is 16% for alcoholics and nearly 30% for alcoholics with liver diseases [5]. Second, the presence of HCV infection correlates with the severity of the disease in alcoholic subjects, that is, HCV-infected patients with ALD develop liver cirrhosis and HCC at a significantly younger age than uninfected ALD patients, suggesting that alcohol and HCV work synergistically to cause liver damage [6]. Many studies also support synergistic interactions between HCV and alcoholism in hepatocarcinogenesis [7–11]. Heavy alcohol consumption and viral hepatitis synergistically increase the risk for HCC among blacks and whites in the U.S. [10]. HCC odds ratio increases to 48.3-fold and 47.8 from 8.1 and 8.6 by having concomitant alcohol abuse in HBV or HCV infected patients, respectively [10]. Indeed, our recent result demonstrates that the incidence of spontaneous HCC induction in the HCV core transgenic mice is increased 2-fold by chronic alcohol intake.

Recent studies with mice expressing HCV proteins have shed pivotal insights into the mechanisms underlying this synergism. The HCV core protein causes overproduction of reactive oxygen species which appears to be responsible for mitochondrial DNA damage [3, 12, 13]. The core protein also inhibits microsomal triglyceride transfer protein activity and VLDL secretion [14], which may underlie the genesis of fatty liver. The core protein also induces insulin resistance in mice and cell lines, and this effect may be mediated by degradation of insulin receptor substrates (IRS) 1 and 2 via upregulation of SOCS3 [15] in a manner dependent on PA28*γ* 73, or via IRS serine phosphorylation [16]. Thus, these core-induced perturbations such as oxidant stress and insulin resistance, which are also known risk factors for ALD, may underlie the synergism reproduced in alcohol-fed core transgenic mice [17]. TLR2 and TLR4 are markedly upregulated in hepatocytes, Kupffer cells, and peripheral monocytes of patients with chronic hepatitis C. TLR2-mediated activation by hepatitis C is linked to the proinflammatory cytokine induction [18]. TLR-mediated signals result in liver disease associated with hepatitis B, hepatitis C, alcoholicnonalcoholic steatohepatitis, and hepatic fibrosis [19]. The most devastating consequence of the synergism between viral hepatitis and alcohol is HCC [7–11]. The risk of developing HCC as assessed by odds ratio increases from 8~12 to 48~54 by having concomitant alcohol abuse in HCV/HBV infected patients [9, 10]. The HCV core and NS3 protein activate TLR2/TLR1 and TLR2/TLR6 on monocytes to produce inflammatory cytokines [19]. The aforementioned effects of the core protein may contribute to the mechanisms of the synergism. However, the more direct mechanistic evidence has recently been attained by our research using mice expressing the HCV nonstructural protein NS5A in a hepatocyte-specific manner. These mice, when fed alcohol for 12 months, develop liver tumors in a manner dependent on TLR4 induced by NS5A [20]. This NS5A-induced TLR4 is activated by endotoxemia associated with alcohol intake, leading to accentuated TLR4 signaling which in turn upregulates the stem cell marker Nanog required for TLR4-dependent liver oncogenesis. This finding on the NS5A-TLR4-Nanog axis in synergistic oncogenesis, is beginning to shed a novel insight into molecular mechanisms for HCC in alcoholic HCV patients.

HCV contains a 9.5-kb single-stranded positive-sense RNA genome, which encodes a polyprotein that is, processed into multiple proteins by cellular and viral proteases. Nonstructural protein NS5A may interact with an interferon-induced, double-stranded RNA-activated protein kinase PKR [21], thus accounting for the resistance of most HCV strains to interferon treatment. NS5A has also been shown to have a cryptic trans-acting activity for some cellular gene promoters [22]. The core protein deserves special mention because, in addition to being a viral structural protein, it serves multiple regulatory functions, including the activation or suppression of various cellular and viral gene promoters. Furthermore, it binds to LT*β*R, TNF receptor and several other cellular proteins, including apolipoprotein AII [23]. The immune- and cytokine-mediating roles of the HCV core protein may play a key role in the synergistic effects of alcohol liver disease (ALD) on HCV-associated liver damage.

## 2. TLRs Signaling in HCC

The TLR signaling pathway is upregulated in chronic liver diseases. Many different cell types in the liver express TLRs [19]. Hepatocytes express TLR1 through TLR9. Stellate cells express TLR2, 3, and 4. Bile duct epithelium expresses TLR2, 3, 4 and 5. Kupffer cells express TLR2, 3, and 4. Chronic alcohol consumption activates other TLRs, such as TLR1, 2, and 6–9, which further increases the TNF-*α* response to LPS in mice [19]. Human monocytes exposed to ethanol for a week develop hypersensitivity to LPS through decreased IRAK-M expression, which activates mitogen-activated protein kinase (MAPK) and NF-*κ*B through TLR4 signaling, leading to activation of NF-*κ*B, AP-1, and ERK [24].

Hepatocyte-specific deletion of TAK1 in mice causes spontaneous hepatocyte death, inflammation, fibrosis, and carcinogenesis partially mediated by TNFR signaling, indicating that TAK1 is an essential component for cellular homeostasis in the liver. In a NASH mouse model, TLR9 signaling induces production of IL-1*β* by Kupffer cells, leading to steatohepatitis, inflammation, and fibrosis via induction of IL-1*β* [25]. Furthermore, modulation of TGF-*β* signaling by a TLR4-MyD88-NF-*κ*B pathway links between profibrogenic and proinflammatory signals [26].

Several possible mechanisms may explain the high prevalence rate of HCV among alcoholics and the increased severity of liver diseases in these patients. First, alcohol may enhance the replication of HCV and thus increase the expression of viral RNA and proteins, resulting in more severe HCV-induced liver injury, independent of the damage induced by alcohol alone. Indeed, HCV titer has been shown to exhibit a correlation with the amount of alcohol consumption [27]. This enhanced effect on HCV replication could be caused directly by the metabolites of ethanol, such as acetaldehyde and free radicals, which may stimulate HCV replication and gene expression. It could also be caused indirectly through alcohol-induced inhibition of the antiviral immune response. Indeed, HCV replication is more active in immunodeficient patients, such as HIV-infected patients [28], and ethanol consumption can cause immunosuppression [29]. 

Another potential mechanism is the involvement of cytokines. Both ALD and HCV cause enhanced secretion of TNF and other cytokines, such as IL-1, IL-6, and IL-8, [30]. TNF is particularly interesting because there is a tight correlation between the serum TNF concentration and the severity of ALD [31, 32], and TNFR1 deficiency ameliorates experimental ALD [33]. TNF may cause cell death through the activation of the TRADD/FADD signal transduction pathway. Oxidative stress may also contribute to TNF cytotoxicity [34]. On the other hand, a variety of factors can modulate the effects of TNF; for examples, NF-*κ*B [35–37], manganous superoxide dismutase (MnSOD) [38], and GSH inhibit TNF-induced cytotoxicity [39]. In experimental ALD, the mitochondrial pool of GSH is depleted and the hepatocytes become hypersensitive to TNF [39]. We have recently shown that HCV core protein binds to lymphotoxin-*β* receptor and TNF receptor [40] and that the expression of this protein in several cell lines sensitize them to TNF-induced cytolysis [41]; therefore, HCV-infected cells are particularly sensitive to TNF. It is interesting to note that HCV core protein also sensitizes cells to apoptosis mediated by Fas [42], which shares with TNF receptors signal transduction molecules such as FADD. These observations suggest that HCV-infected hepatocytes are very sensitive to TNF and possibly other cytokines as well. This enhanced sensitivity, coupled with the increased secretion of TNF in ALD, may account for the synergistic effects of ALD on HCV.

## 3. TLR4-Mediated AP1 Activation and HCC

Alcoholism is associated with endotoxemia that stimulates expression of proinflammatory cytokine expression and inflammation in the liver and fat tissues [43]. Development of liver cancer in the HCV core mice is associated with inflammation [44]. Expression of the proinflammatory cytokine TNF-*α* and IL-1*β* is induced by HCV-infected human B cells (16) and by its core protein in the transgenic mouse model [45, 46]. Recently, we have shown that HCV infection, through NS5A protein expression, upregulates TLR4 expression and proinflammatory cytokines [47], providing a potential explanation for increased inflammation in HCV-infected livers. Further accentuation of TLR4 signaling in HCV would be expected if combined with alcohol abuse. This may serve as part of the synergistc mechanism when superimposed by other key patholophysiological events common in these comorbidities such as CYP2E1 induction. CYP2E1 induction impairs hepatic insulin signaling [48, 49], induces oxidative DNA damage [50], primes macrophages to increase LPS-induced TNF-*α* production [51], sensitizes hepatocytes to TNF-*α*-mediated cell death via c-jun [52, 53], and more importantly, leads to marked potentiation of endotoxin-induced oxidant liver injury (35). Therefore, saturated fatty acids which are implicated in obesity and diabetes could serve as additional ligands to enhance signaling via TLR4 which is already upregulated by HCV NS5A [54]. Polyunsaturated fats actually result in greater rather than less liver injury [55]. These interactive and synergistic mechanisms involving TLR4 in HCV and alcohol most likely contribute to increased incidence of HCC via oxidant stress and inflammation and are the focal points of my proposed research. 

c-Jun-deficient mice die between embryonic days E12.5 and E13.5 from massive apoptosis of hepatoblasts, erythroblasts, and other cell types [56, 57]. To overcome this problem, mice harboring a “floxed” c-jun allele that can be deleted in designated cell types upon expression of the Cre recombinase have been developed. Using this system, c-Jun expression was shown to be essential for proper proliferation in postnatal hepatocytes [58]. Moreover, the deletion of c-Jun in hepatocytes compromises the ability of these cells to enter the cell cycle and undergo rapid proliferation after partial hepatectomy [58]. A well-accepted model of HCC utilizes a chemical carcinogen DEN (diethylnitrosamine) as a tumor initiator and phenobarbital as a promoter. By using this model and tissue-specific knockout mice, the loss of JNK1 in the liver was shown to reduce DEN-induced HCC development [59]. The requirement for c-*jun* was restricted to an early stage of tumor development in chemically induced HCC in mice [60]. In our study, c-jun knockout dramatically reduced the incidence of spontaneous and DEN-induced HCC in HCV core transgenic mice, supporting the role of c-jun in this model of hepatocarcinogenesis (Hepatology in press). Alcoholic liver disease patients have increased levels of hepatic RANTES/CCL5. Ethanol augments RANTES/CCL5 expression in rat liver sinusoidal endothelial cells and human endothelial cells via activation of NF-*κ*B, HIF-1*α*, and AP-1 [61].* In vitro* studies using liver-derived cell lines have demonstrated rapid activation of AP-1 by HBV or HCV proteins [62], and this mitogenic effect is implicated in hepatocytes' susceptibility to liver cell transformation via fixation of genetic mutations caused by oxidant stress. Indeed, Further c-Jun prevents apoptosis by antagonizing p53 activity as another contributing factor for HCC development [60, 63]. Ectopic expression of HCV core protein constitutively activates AP-1 via JNK [64]. Activation of JNK is also implicated in ASH and NASH [49, 52]. Therefore, activation of JNK and c-jun most likely plays pivotal roles in synergistic induction of liver cancer by HCV and alcohol.

### 3.1. Cancer Stem Cells and HCC

Stem cells have three major characteristics: self renewal, asymmetric and multiple cell division (clonality), and plasticity. The liver has a high regenerative potential, and hepatic small oval progenitor cells around the peripheral branches of the bile ducts, the canals of Hering, can differentiate into biliary epithelial cells and hepatocytes [65]. These oval liver progenitor cells share molecular markers with adult hepatocytes (albumin, cytokeratin 7 [CK7], CK19, oval cell markers (OV-6, A6, and OV-1), chromogranin-A, NCAM (neural cell adhesion molecule)) and fetal hepatocytes (*α*-fetoprotein) (Table 1) [65, 66]. They are also positive for more common stem cell markers such as CD34+, Thy-1+, c-Kit+, and Flt-3+ (FMS-like tyrosine kinase 3) [67]. Thus, it currently remains unclear whether these stem cells are derived from the bone marrow and just migrate to this periportal niche or whether they represent true resident liver stem/progenitor cells. Binding of stroma-derived factor-1*α* (SDF-1*α*) to its surface receptor CXCR4 activates oval hepatic cells [68]. Forty percent of HCC have clonality, and thus are considered to originate from progenitor/stem cells [66, 69–71]. Recent studies of HCC have centered on CSC, including detection of CSC in cancer, identification of CSC markers, and isolation of CSC from human HCC cell lines. CSC were identified as a CD117+/CD133+ hepatic precursors in regenerating liver tissue [72] and a CD45−/CD90+ subpopulation of tumor cells in HCC [73]. The CD90+ cells are not present in the normal liver and, when injected into immunodeficient mice, create tumors repeatedly. In human HCC and HCC cell lines, specifically, CD133+ cells, not CD133− cells, had the ability to self-renew, create differentiated progenies, and form tumors [74]. This coincided with the expression of genes associated with stem/progenitor status, such as *β*-catenin, NOTCH, BMI, and OCT3/4. When compared to CD133− cells, the CD133+ cells isolated from the HCC cell lines showed higher expression of CD44 and CD34, but both CD133 subpopulations displayed similar expression for CD29, CD49f (integrin *α*6), CD90 and CD117 [74]. Furthermore, CD133+/CD49f+ cells in liver tumors correlate with tumorigenecity and “stemness” genes, such as Wnt/*β*-catenin, Notch, Hedgehog/SMO, Bmi, and Oct3/4 [75–77]. CD133+/CD49f+ HCC cancer stem cells confer resistance to chemotherapy, and this presents a major obstacle for the treatment of HCC [78]. One potential reason for this chemoresistance may lie in the plasticity of cancer stem cells with dysregulated signaling and gene expression. Several oncogenic signaling pathways in cancer stem cells of HCC, have been described including activated PI3K/AKT [79], signal transducer and activator of transcription 3 (STAT3) [80, 81], Notch [82], Hedgehog [83, 84], and transforming growth factor-beta (TGF-*β*) [85, 86].

Nanog is one of the core transcription factors found in pluripotent embryonic stem cells (ESCs) [95]. It is essential for maintaining self-renewal and pluripotency of both human and mouse embryonic stem cells [96–99]. Overexpression of Nanog induces and maintains the pluripotency and self-renewing characteristics of ESCs under what normally would be differentiation-inducing culture conditions [100]. Recently, Nanog expression has been reported in human neoplasms, including germ cell tumors [101–104], breast carcinomas [104], osteosarcoma [105], and HCC [79]. Ectopic expression of Nanog induces an oncogenic potential in NIH3T3 [106].

## 4. Nanog-Positive Cancer Stem Cells Induced by HCV and Alcohol

Alcohol synergistically enhances the progression of liver disease and the risk for liver cancer caused by HCV. Toll-like receptor 4 (TLR4) is induced by hepatocyte-specific transgenic (Tg) expression of the HCV nonstructural protein NS5A, and this induction mediates synergistic liver damage and tumor development by alcohol-induced endotoxemia [20]. The stem/progenitor cell marker, Nanog, is upregulated as a novel downstream gene by TLR4 activation and the presence of CD133/Nanog-positive cells in liver tumors of alcohol-fed NS5A Tg mice [20]. Transplantation of p53-deficient hepatic progenitor cells transduced with TLR4 results in liver tumor development in mice following repetitive lipopolysaccharide (LPS) injection, but concomitant transduction of Nanog short-hairpin RNA abrogates this outcome [20]. Despite the common understanding that TLR4 is one of the pattern recognition receptors expressed predominantly by innate immune cells such as macrophages and lymphocytes, our study demonstrates that hepatocytes can be the primary cellular site of both TLR4 upregulation and its pathologic consequences in the context of HCV infection. Therefore, the TLR4-dependent mechanism synergizes liver disease by HCV and alcohol and is partly dependent on Nanog, a TLR4 downstream gene.

Nanog transduction alone is not as effective as TLR4 activation in liver tumorigenesis, as shown by our cell transplantation experiment [20]. We believe that TLR4 activation induces other tumor-driver genes which cooperatively work with Nanog to cause liver oncogenesis. Thus, Nanog is still essential for TLR4-dependent oncogenesis, but it alone is poorly oncogenic. In our previous work using a cell line, we demonstrated that TLR4 promoter up-regulation by NS5A is mediated by PU.1, Oct-1, and AP-1 elements [47]. The similar transcriptional mechanism may underlie TLR4 induction in primary hepatocytes.

In summary, alcohol and HCV NS5A synergistically induce liver tumor development via induction and activation of TLR4 in mice. The importance of Nanog as a direct downstream gene of TLR4 in this oncogenesis has also been identified. Pharmacologic inhibition of TLR4 signaling may become a novel therapeutic strategy for HCV-associated liver tumors. 

## Figures and Tables

**Table 1:** Markers for liver cancer stem cells.

Gene name	Other name	Function	Species	Organ	References
CD133	Prominin 1 (PROM1)	Glycoprotein, membrane protrusions	Human, mouse	Liver, brain	[74, 78, 79, 87–89]
CD49f	Integrin*α* chain *α*6 (ITGA6)	Cell adhesion, cell signaling	Mouse	Liver	[74, 78]
CD90	Thy-1	Glycophosphatidylinositol (GPI) anchor	Mouse	Liver	[74]
CD44	Hyaluronic acid receptor	Cell adhesion and migration, metastasis	Mouse	Liver, breast	[74, 90]
CD117	KIT	C-kit receptor cytokine receptor	Mouse	Liver	[74]
CK19	Cytokeratin 19	Biliary lineage marker	Mouse	Liver	[91, 92]
OV-6	Oval cell marker	Early progenitor cells	Human	Liver	[92]
CD34	Glycoprotein	Cell-cell adhesion factor	Mouse	Liver, leukemia	[93]
AFP	*α*-fetoprotein	Fetal counterpart of serum albumin	Mouse	Liver	[94]
